# Oxygen-Vacancy Engineered SnO_2_ Dots on rGO with N-Doped Carbon Nanofibers Encapsulation for High-Performance Sodium-Ion Batteries

**DOI:** 10.3390/molecules30153203

**Published:** 2025-07-30

**Authors:** Yue Yan, Bingxian Zhu, Zhengzheng Xia, Hui Wang, Weijuan Xu, Ying Xin, Qingshan Zhao, Mingbo Wu

**Affiliations:** 1 State Key Laboratory of Heavy Oil Processing, Shandong Key Laboratory of Advanced Electrochemical Energy Storage Technologies, College of Chemistry and Chemical Engineering, China University of Petroleum (East China), Qingdao 266580, China; 15940535308@163.com (Y.Y.); zhubingxian0909@163.com (B.Z.); xiazz2021@163.com (Z.X.); 17613877583@163.com (H.W.); 19861453181@163.com (W.X.); 18364538700@163.com (Y.X.); wumb@upc.edu.cn (M.W.); 2 College of Chemical Engineering, Qingdao University of Science & Technology, Qingdao 266100, China

**Keywords:** oxygen-vacancy engineering, reduced graphene oxide, SnO_2_ dots, hierarchical structure, sodium-ion battery

## Abstract

The widespread adoption of sodium-ion batteries (SIBs) remains constrained by the inherent limitations of conventional anode materials, particularly their inadequate electronic conductivity, limited active sites, and pronounced structural degradation during cycling. To overcome these limitations, we propose a novel redox engineering approach to fabricate oxygen-vacancy-rich SnO_2_ dots anchored on reduced graphene oxide (rGO), which are encapsulated within N-doped carbon nanofibers (denoted as ov-SnO_2_/rGO@N-CNFs) through electrospinning and subsequent carbonization. The introduction of rich oxygen vacancies establishes additional sodium intercalation sites and enhances Na^+^ diffusion kinetics, while the conductive N-doped carbon network effectively facilitates charge transport and mitigates SnO_2_ aggregation. Benefiting from the well-designed architecture, the hierarchical ov-SnO_2_/rGO@N-CNFs electrode achieves remarkable reversible specific capacities of 351 mAh g^−1^ after 100 cycles at 0.1 A g^−1^ and 257.3 mAh g^−1^ after 2000 cycles at 1.0 A g^−1^ and maintains 177 mAh g^−1^ even after 8000 cycles at 5.0 A g^−1^, demonstrating exceptional long-term cycling stability and rate capability. This work offers a versatile design strategy for developing high-performance anode materials through synergistic interface engineering for SIBs.

## 1. Introduction

The widespread adoption of lithium-ion batteries (LIBs) across green energy storage, electric vehicles, and mobile electronics stems from their exceptional energy storage capacity and reliable long-term cyclability. However, the limited global lithium resources and high costs necessitate the exploration of alternative energy storage systems. Among these alternatives, sodium-ion batteries (SIBs) have emerged as a promising alternative, owing to their cost-effectiveness, naturally abundant resources, and improved safety features [1,2]. Despite these advantages, most SIB electrode materials suffer from low specific capacity and low redox potential, which hinders the effective improvement of energy density [3]. As a result, the expected cost advantages of SIBs may not be fully realized. Therefore, advancing electrode materials with superior energy density represents a critical step toward the commercial viability of SIBs. In particular, developing high-performance anodes for SIBs remains a critical challenge, as traditional graphite anodes used in LIBs exhibit poor sodium storage capacity.

Tin dioxide (SnO_2_) has emerged as a potential anode material because of its remarkably high theoretical storage capacity (667 mAh g^−1^), low discharge potential, low cost, and natural abundance [4,5]. However, during the sodium storage process, SnO_2_ undergoes a two-step reaction comprising initial formation of inactive Na_2_O and active Na_x_Sn alloy. This reaction results in low conductivity, sluggish kinetics, and significant volume expansion, ultimately causing rapid capacity fading within the initial cycles [6]. Incorporating a conductive carbon framework effectively mitigates these issues by inhibiting metal oxide particle aggregation while improving the overall charge transport properties of the electrode material, thus improving electrochemical performance. Various carbon materials, such as graphene [7], porous carbon [8], carbon nanotubes [9], and carbon nanofibers [10], have been successfully combined with SnO_2_ to enhance its electrochemical properties. Wang et al. established a facile route for preparing SnO_2_ nanoflake arrays on carbon cloth, subsequently modified with polypyrrole (PPy) through electrodeposition [11]. The flexible SnO_2_@PPy electrode exhibited outstanding cycling stability and rate performance. Similarly, Yang et al. introduced a template-independent “heterogeneous carbonization” approach to synthesize SnO_2_/carbon/void/carbon (SCVC) nanofibers, subsequently combining them with rGO as a conductive additive to fabricate a self-supporting SCVC-rGO anode. The prepared anode composite demonstrated superior sodium storage performance, including exceptional reversible capacity, extended cycle stability, and outstanding rate characteristics [12,13].

Compared with surface modifications such as carbon compositing, which do not fundamentally alter the atomic or electronic structure of the host material, defect engineering of SnO_2_ can effectively reduce diffusion energy barriers, create additional intercalation sites, and enhance electrode kinetics. These improvements collectively contribute to enhanced electrochemical performance [14,15]. For example, Yang et al. developed biomass-derived N-doped carbon microspheres embedded with SnO_2_ nanoparticles containing numerous oxygen defects (SNC composites). This architecture maintained excellent long-term cyclability, maintaining 320 mA·h·g^−1^ at 1 A·g^−1^ after 1000 cycles [16]. Ma et al. developed an effective approach to synthesize carbon nanofibers embedded with SnO_2−x_ nanoparticles featuring oxygen vacancies, demonstrating superior reversible capacity and exceptional cycling durability as an anode material [17]. Xu et al. employed AAO template-directed assembly coupled with ALD to fabricate well-ordered amorphous SnO_2_ architectures. This oxygen-vacancy-rich electrode material exhibited excellent capacity retention, delivering 376 mAh g^−1^ after 100 cycles at low current (0.05 A g^−1^) and 220 mAh g^−1^ following 800 cycles at a high rate (1 A g^−1^) [18]. Despite the promising electrochemical performance of oxygen-vacancy SnO_2_ reported in these studies, large SnO_2_ particles still face challenges such as limited cycle life at high current densities [19]. In addition, challenges such as complex synthesis procedures and uncontrolled particle growth of oxygen-vacancy-rich SnO_2_ warrant further investigation.

Recent studies have demonstrated the great promise of electrospun Sn-based composites in enhancing the sodium storage performance, due to their tunable 1D nanostructures, mechanical flexibility, and ability to accommodate volume changes. For example, Zhou et al. prepared a SnO_2_/C composite nanofiber via electrospinning, achieving excellent cycling stability and rate capability [20]. Electrospinning engineering has also been widely applied to design hierarchical nanostructures with well-dispersed Sn-based components [21]. Moreover, MOF-derived electrospun materials have attracted growing attention for their structural versatility and metal content control [22].

Building upon these advancements, we propose an effective strategy for fabricating oxygen-vacancy-rich SnO_2_ dots anchored on rGO and encapsulated within N-doped carbon nanofibers (ov-SnO_2_/rGO@N-CNFs) via electrospinning and carbonization. In this approach, GO (graphene oxide) serves as both an oxidizing agent and a structure-directing component, facilitating a rapid redox reaction with Sn^2+^ precursors to enable the formation of ultrasmall SnO_2_ dots (~2.3 nm) with abundant oxygen-vacancy defects. The synergistic integration of oxygen-deficient SnO_2_ dots, conductive rGO network, and protective N-doped carbon nanofibers (N-CNFs) establishes a hierarchical architecture to achieve additional sodium intercalation sites, enhanced Na^+^/electron transport, and buffered SnO_2_ aggregation. Consequently, the ov-SnO_2_/rGO@N-CNFs electrode demonstrates remarkable electrochemical performance, including high reversible capacity and exceptional long-term cycling stability, validating the effectiveness of this multifunctional design approach for advanced SIB anodes.

## 2. Results and Discussion

**Figure 1:**
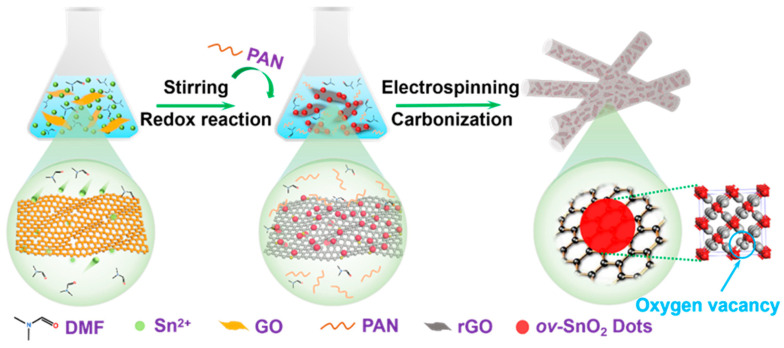
Schematic illustration for the synthetic process of ov-SnO_2_/rGO@N-CNFs.

The abundant oxygen-containing functional groups in GO enable effective oxidation of metal ions while maintaining strong electrostatic interactions [23]. Therefore, the fabrication strategy leverages the unique dual functionality of GO, which serves as both a mild oxidizing agent and a structural template. Figure 1 illustrates the synthesis pathway, where a spontaneous redox reaction between GO and Sn^2+^ ions results in the uniform deposition of highly dispersed SnO_2_ dots (~2.3 nm) on rGO nanosheets. This process not only ensures the homogeneous distribution of SnO_2_ but also induces controlled defect engineering through incomplete crystallization, generating a high concentration of oxygen vacancies (OVs). To address the intrinsic volume expansion challenge of SnO_2_ during sodiation/desodiation cycles, a dual-confinement approach was implemented through electrospinning and subsequent carbonization. The SnO_2_/rGO-DMF intermediate becomes encapsulated within PAN during electrospinning, forming, after carbonization, a robust hierarchical structure where SnO_2_ dots are mechanically stabilized between conductive rGO nanosheets and N-CNFs.

**Figure 2:**
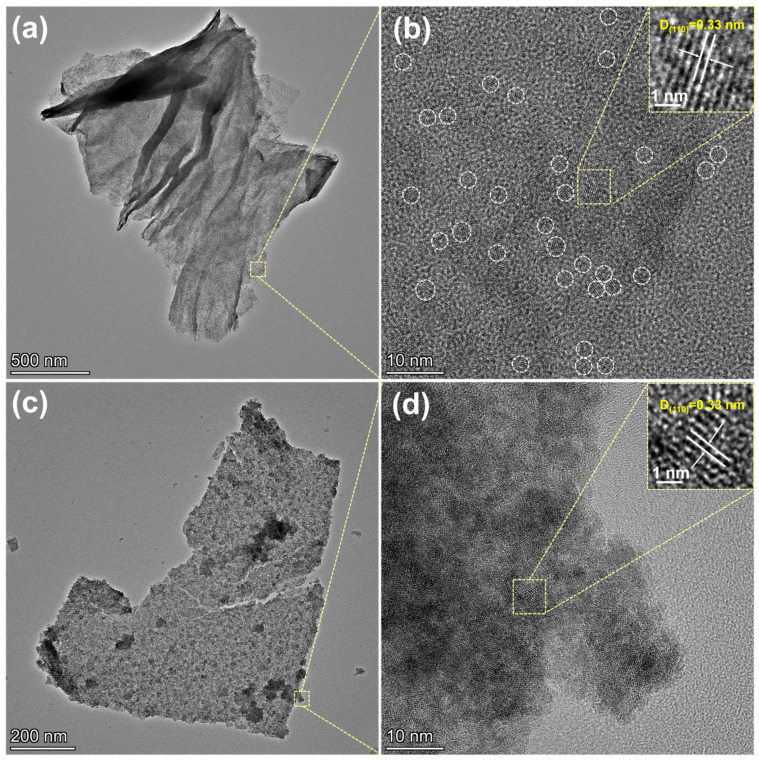
TEM images of (**a**,**b**) SnO_2_/rGO-DMF and (**c**,**d**) SnO_2_/rGO-H_2_O.

The dispersion characteristics of SnO_2_ prepared in different solvent systems were systematically investigated through TEM analysis. Figure 2 presents a comparison between SnO_2_/rGO composites synthesized in DMF (Figure 2a,b) and H_2_O (Figure 2c,d) solvents. The SnO_2_/rGO-DMF sample exhibits superior dispersion of ultra-small SnO_2_ nanoparticles uniformly distributed across the rGO flakes, while the aqueous system shows significant particle aggregation that obscures clear size determination. High-resolution structural characterization confirms crystalline SnO_2_ formation in each sample, exhibiting distinct lattice fringes measuring 0.33 nm that match the (110) crystallographic orientation. The observed solvent-dependent morphology differences demonstrate that organic solvents generally produce smaller and more uniformly dispersed nanoparticles compared to aqueous systems [24,25,26]. The observed behavior primarily stems from the strong dipole moment characteristic of DMF, which modifies the surface Gibbs free energy during low-temperature nanocrystal growth and effectively inhibits interparticle contact and coalescence [27,28,29]. Further XRD analysis (Figure S1) confirms the successful formation of SnO_2_ in both samples, with diffraction patterns matching the standard cassiterite structure (JCPDS: 41-1445). However, significant differences in peak broadening are evident among the samples. Both SnO_2_/rGO composites show broader and weaker diffraction peaks compared to pristine SnO_2_, with the DMF-derived sample exhibiting the most pronounced peak broadening. This observation is consistent with the TEM findings, further verifying the reduced crystallite size and improved dispersion in SnO_2_/rGO-DMF [30].

**Figure 3:**
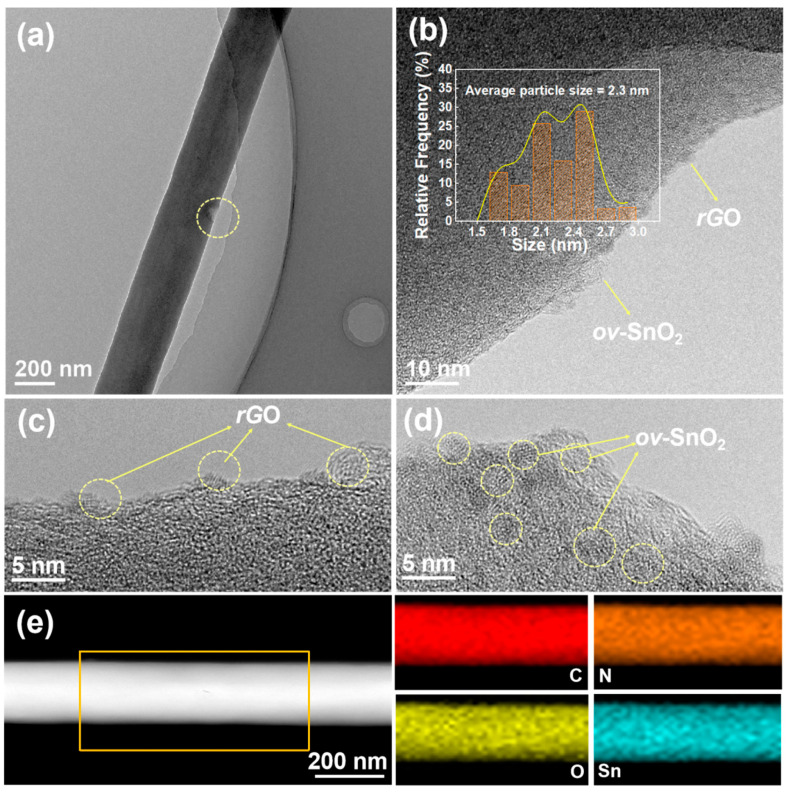
(**a**) TEM images of ov-SnO_2_/rGO@N-CNFs. (**b**) HRTEM image and particle size distribution (the inset) of ov-SnO_2_/rGO@N-CNFs. (**c**,**d**) HRTEM image of rGO and ov-SnO_2_ in ov-SnO_2_/rGO@N-CNFs. (**e**) EDX elemental mapping of ov-SnO_2_/rGO@N-CNFs.

The SnO_2_/rGO-DMF composite was further processed through electrospinning with PAN coating followed by carbonization to produce the final ov-SnO_2_/rGO@N-CNFs. SEM and TEM characterization (Figure S2 and Figure 3a,b) reveal the detailed microstructure of this composite, showing uniformly dispersed ultrafine nanodots averaging 2.3 nm within the carbon nanofibers without obvious particle aggregation. Close examination of the bare fiber edges in Figure 3c identifies the presence of rGO nanoflakes, while Figure 3d demonstrates that the SnO_2_ dots lack clearly visible lattice fringes, indicating their low crystallinity. This hierarchical structure effectively utilizes rGO substrates and N-CNFs to prevent SnO_2_ aggregation, thereby maintaining structural integrity even under high current density cycling conditions. The elemental mapping in Figure 3e confirms the uniform spatial dispersion of C, N, O, and Sn throughout the material, providing direct evidence for the uniform dispersion of ultrafine SnO_2_ within the N-doped carbon nanofiber matrix. XRD analysis (Figure S3) reveals that carbonized-SnO_2_/rGO-DMF shows sharp diffraction peaks due to SnO_2_ aggregation and crystallization during high-temperature treatment, while ov-SnO_2_/rGO@N-CNFs exhibits only a broad hump around 25° without distinct peaks, suggesting effective confinement of small, low-crystallinity SnO_2_ dots within the carbon nanofibers. Notably, control experiments without GO (Sn@N-CNFs) resulted in the formation of metallic Sn during carbonization, in agreement with prior studies [31], highlighting the essential function of GO in preserving the oxide structure under these synthetic conditions.

**Figure 4:**
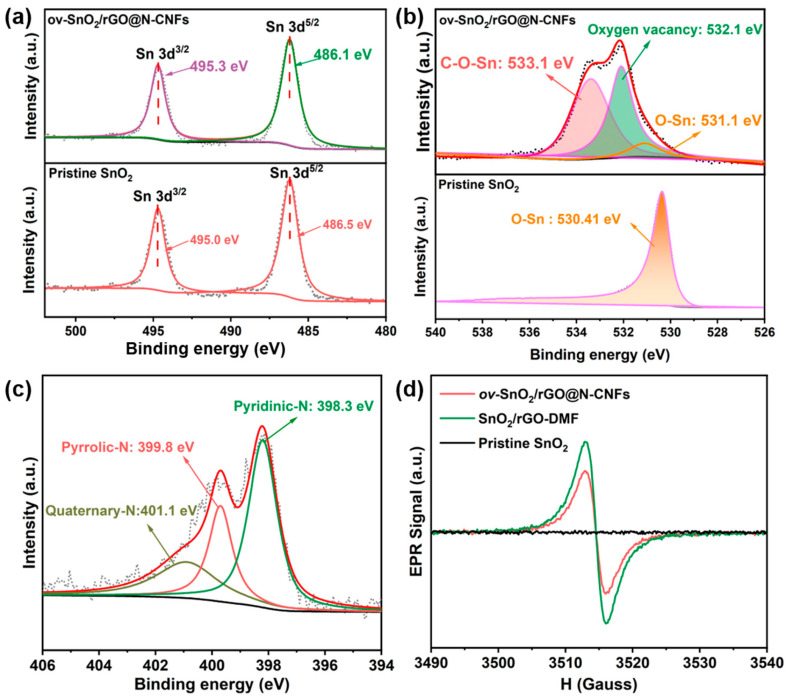
(**a**) Sn 3d of ov-SnO_2_/rGO@N-CNFs and pristine SnO_2_, (**b**) O 1s of ov-SnO_2_/rGO@N-CNFs and pristine SnO_2_, (**c**) N 1s XPS spectra of ov-SnO_2_/rGO@N-CNFs. (**d**) EPR profiles of ov-SnO_2_/rGO@N-CNFs, SnO_2_/rGO-DMF, and pristine SnO_2_.

The chemical composition and surface characteristics of ov-SnO_2_/rGO@N-CNFs were thoroughly investigated through XPS analysis. The Sn 3d XPS spectra (Figure 4a) reveal distinct peaks at 495.0 eV (3d_3/2_) and 486.0 eV (3d_5/2_) for both ov-SnO_2_/rGO@N-CNFs and pristine SnO_2_, with the composite material exhibiting a noticeable shift toward lower binding energies. This shift suggests significant electronic structure modifications, potentially arising from multiple factors, including the existence of oxygen defects along with partial conversion to Sn^2+^ states or interfacial charge transfer between SnO_2_ and the carbon matrix [19,32]. Further examination of the O 1s spectrum (Figure 4b) identifies peaks of C-O-Sn at 533.1 eV, O-Sn at 531.1 eV, and a characteristic peak at 532.1 eV corresponding to oxygen vacancies [31,33]. Deconvolution of the N 1s XPS profile (Figure 4c) demonstrates three distinct nitrogen configurations, including pyridinic-N (398.3 eV), pyrrolic-N (399.8 eV), and quaternary N (401.1 eV). With a Sn loading of 21.68 wt% (Table S1), the material exhibits a favorable combination of active sites and conductivity, as evidenced by its high reversible capacity and stable cycling performance. Notably, the presence of pyridinic-N and quaternary nitrogen in sp^2^ hybridization configuration substantially improves charge transport properties within the carbon framework, thereby contributing to improved electrochemical performance [34,35]. Complementary EPR analysis (Figure 4d) provides additional evidence for oxygen vacancies, showing a pronounced signal at g = 2.003 for both ov-SnO_2_/rGO@N-CNFs and SnO_2_/rGO-DMF, while pristine SnO_2_ displays no such signal. The EPR signal at g = 2.003 is attributed to unpaired electrons associated with oxygen vacancies generated within the SnO_2_ lattice as a result of GO-induced redox processing. The slightly reduced signal intensity in ov-SnO_2_/rGO@N-CNFs compared to SnO_2_/rGO-DMF likely results from partial shielding of defect sites by the carbon fiber coating. These findings collectively demonstrate that GO not only facilitates the formation of ultrasmall SnO_2_ particles but also promotes oxygen-vacancy generation. Remarkably, the oxygen-vacancy-rich defect structure is retained even after the high-temperature carbonization process.

**Figure 5:**
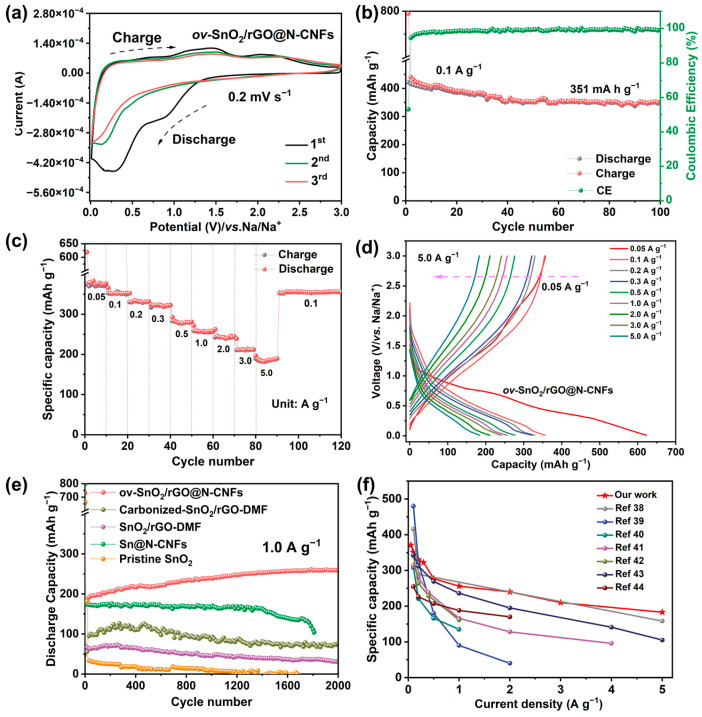
(**a**) CV curves of ov-SnO_2_/rGO@N-CNFs at 0.2 mV·s^−1^. (**b**) Cycling performances of ov-SnO_2_/rGO@N-CNFs at 0.1 A·g^−1^. (**c**) Rate capability of ov-SnO_2_/rGO@N-CNFs at different current densities. (**d**) Corresponding galvanostatic discharge/charge profiles at different current densities. (**e**) Cycling performance of ov-SnO_2_/rGO@N-CNFs, SnO_2_/rGO-carbonization, SnO_2_/rGO-DMF, Sn@N-CNFs and pristine SnO_2_ at 1.0 A·g^−1^. (**f**) Rate capacity comparison of ov-SnO_2_/rGO@N-CNFs with previously reported SnO_2_ electrodes [38,39,40,41,42,43,44].

Cyclic voltammetry and galvanostatic charge-discharge measurements were employed to comprehensively assess the electrochemical behavior of the ov-SnO_2_/rGO@N-CNFs hybrid material. Figure 5a presents the initial three CV cycles measured between 0.01–3.0 V (vs Na^+^/Na) at 0.2 mV s^−1^, revealing distinct redox processes. During the initial cathodic sweep, a distinct reduction feature appears between 0.85–1.0 V, attributed to the stepwise transformation of SnO_2_ into metallic Sn (via SnO intermediate) coupled with solid electrolyte interphase (SEI) layer formation (SnO_2_ + 2Na^+^ + 2e^−^ → SnO + Na_2_O and SnO + 2Na^+^ + 2e^−^ → Sn + Na_2_O) [19,33]. An additional reduction feature appears at 0.0–0.5 V, attributed to the alloying reaction (Sn + xNa^+^ + xe^−^ → Na_x_Sn, 0 ≤ x ≤ 3.75) [13,31]. Subsequent anodic scans exhibit oxidation peaks at 0.6 V, 1.45 V, and 2.0 V, representing the stepwise reconversion processes. Galvanostatic cycling at 0.1 A g^−1^ (Figure 5b) demonstrates an initial discharge capacity of 795.2 mAh g^−1^ with 53.1% coulombic efficiency (CE), reflecting inevitable SEI formation and electrolyte decomposition. The efficiency rapidly stabilizes above 99% after a few cycles, delivering a maintained capacity of 351 mAh g^−1^ after 100 cycles. This performance is in line with the calculated capacity of 340.7 mAh g^−1^ (Table S2), estimated based on the theoretical capacity of SnO_2_ and the experimentally measured capacity of rGO@N-CNFs (Figure S5). The excellent capacity utilization of SnO_2_ can be attributed to its engineered oxygen vacancies, which not only provide additional sodium intercalation sites but also reduce the diffusion barrier for ion transport. Moreover, the ov-SnO_2_/rGO@N-CNFs composite exhibits exceptional rate capability, delivering reversible capacities of 374, 350, 331, 320, 281, 256, 243, 211, and 179 mAh g^−1^ at progressively increased current densities from 0.05 to 5.0 A g^−1^ (Figure 5c,d). The capacity fully recovers to 351 mAh g^−1^ when returning to 0.1 A g^−1^, demonstrating outstanding structural stability. The sloping voltage profiles without distinct plateaus suggest solid-solution type Na^+^ insertion/extraction behavior with continuous phase transitions [36,37], characteristic of the ultrasmall SnO_2_ dots uniformly dispersed within the conductive rGO and N-CNFs matrix. This architecture enables both high capacity retention and superior rate performance by facilitating charge transfer while accommodating volume changes during cycling.

**Figure 6:**
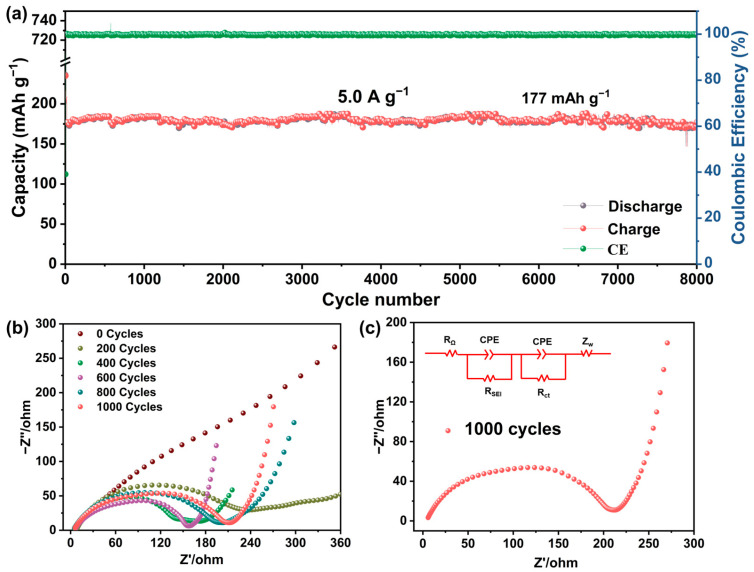
(**a**) Long-term cycling performance of ov-SnO_2_/rGO@N-CNFs at 5.0 A g^−1^. (**b**) The electrochemical impedance spectra of ov-SnO_2_/rGO@N-CNFs after different cycle testing at 1.0 A·g^−1^. (**c**) Nyquist plots (red dots) and equivalent circuit of ov-SnO_2_/rGO@N-CNFs after 1000 cycles.

The long-term cycling stability of various samples was systematically evaluated at 1.0 A g^−1^, as shown in Figure 5e. The ov-SnO_2_/rGO@N-CNFs composite demonstrates exceptional cycling performance, maintaining a capacity of 257.3 mAh g^−1^ after 2000 cycles with a high CE of 99.5%. This performance significantly outperforms the control samples of carbonized-SnO_2_/rGO-DMF (87 mAh g^−1^) and SnO_2_/rGO-DMF (46 mAh g^−1^), while pristine SnO_2_ suffers rapid degradation to merely 31 mAh g^−1^ within 50 cycles. Notably, the Sn@N-CNFs sample exhibits a sharp capacity decline after 1700 cycles, which can be attributed to progressive agglomeration of SnO_2_ during cycling, reducing available active sites for Na^+^ storage. Moreover, particle pulverization during repeated sodiation/desodiation processes also results in loss of electrical contact between SnO_2_ crystallites. Post-cycling TEM analysis (Figure S4a) confirms the structural integrity of ov-SnO_2_/rGO@N-CNFs, with SnO_2_ dots remaining well-dispersed within the carbon nanofiber matrix, while Sn@N-CNFs (Figure S4b) show severe structural collapse due to unmitigated volume changes during alloying/dealloying reactions. As shown in Figure 5f, compared with the reported SnO_2_-based electrodes, ov-SnO_2_/rGO@N-CNFs demonstrates superior rate capability, particularly at high current densities, revealing its outstanding electrochemical performance. To further demonstrate the exceptional long-term cycling stability and structural integrity of the ov-SnO_2_/rGO@N-CNFs composite under extreme conditions, its electrochemical performance was evaluated at an ultrahigh current density of 5.0 A·g^−1^. As shown in Figure 6a, the ov-SnO_2_/rGO@N-CNFs electrode maintains an impressive reversible capacity of 177 mAh g^−1^ after 8000 cycles, demonstrating unprecedented durability for SnO_2_-based anodes. The outstanding electrochemical properties primarily stem from two structural advantages: the atomic-scale SnO_2_ quantum dots that significantly reduce Na^+^ migration pathways, and the abundant redox-active sites facilitating efficient sodium ion intercalation/deintercalation processes. This unique architecture collectively contributes to the superior battery performance observed.

To further elucidate the electrochemical kinetics of the ov-SnO_2_/rGO@N-CNFs electrode, electrochemical impedance spectroscopy (EIS) was performed after various cycling intervals. As illustrated in Figure 6b and Table S3, the Nyquist plots after the 0th, 200th, 400th, 600th, 800th, and 1000th cycles at 1.0 A·g^−1^ exhibit a typical semicircle in the high-to-medium frequency region and a linear tail at low frequencies, corresponding to the charge transfer resistance (R_ct_) and sodium ion diffusion resistance, respectively. The initial decrease in R_ct_ is attributed to electrode activation, while the gradual increase after 400 cycles suggests partial structural degradation, though the overall stability remains superior. The fitted parameters of the 1000th cycles based on the equivalent circuit were displayed in Figure 6c.

Generally, R_s_ represents the cumulative ohmic resistance from the electrode assembly, electrolyte solution, and current-collecting components; R_ct_ corresponds to the energy barrier for charge transfer across the electrode-electrolyte interface; CPE accounts for the non-ideal capacitive behavior arising from surface inhomogeneity; while Z_w_ characterizes the diffusion-controlled impedance associated with sodium ion transport through the electrode matrix. This comprehensive set of parameters provides fundamental insights into the various resistive and capacitive processes occurring within the electrochemical system. It is found that the R_ct_ (3.7 Ω) value at the 1000th cycle is smaller than that at the 0th cycle (R_ct_ = 22.4 Ω) and the 200th cycle (R_ct_ = 16.3 Ω), indicating progressive electrode activation and improved interfacial kinetics. This reduction in R_ct_ suggests that the conductive carbon framework and uniformly dispersed SnO_2_ nanodots effectively facilitate electron transfer at the electrode–electrolyte interface. The initial improvement should be due to enhanced electrolyte penetration and particle size reduction during activation, followed by modest deterioration from partial structural changes while maintaining overall stability [32,45].

Furthermore, the sodium ion diffusion coefficient (D_Na+_), a key kinetic parameter for assessing electrochemical behavior, was determined through analysis of the Warburg impedance region in EIS measurements. Accordingly, the D_Na+_ of ov-SnO_2_/rGO@N-CNFs after 400 °C, 800 °C, and 1000 °C are determined to be 1.32 × 10^−13^ cm^2^ s^−1^, 1.55 × 10^−13^ cm^2^ s^−1^, and 3.02 × 10^−13^ cm^2^ s^−1^, respectively. This progressive enhancement in ionic transport kinetics, coupled with the low and stable charge-transfer resistance, provides fundamental insight into the composite’s exceptional rate capability and long-term cyclability. The combined EIS analysis confirms that the hierarchical architecture of ov-SnO_2_/rGO@N-CNFs successfully maintains favorable charge transport characteristics throughout extended cycling, explaining its superior performance compared to conventional SnO_2_-based anodes. This continuous improvement can be attributed to the well-preserved porous network and oxygen-vacancy-rich structure, which offer fast ion migration pathways and minimize diffusion barriers.

Building upon the insights gained from the EIS analysis, a detailed investigation into the sodium storage mechanism was conducted to better understand the origin of the superior performance. The sodium storage behavior of ov-SnO_2_/rGO@N-CNFs is primarily governed by the conversion and alloying reactions of SnO_2_. Upon discharge, SnO_2_ is first reduced to metallic Sn, which then forms NaₓSn alloys through reversible alloying reactions. During charging, the alloy dealloys and partially reoxidizes, enabling good reversibility. The ultrafine SnO_2_ nanocrystals confined in the carbon nanofibers help buffer volume expansion and maintain structural integrity throughout cycling. Meanwhile, the rGO-modified N-doped carbon matrix provides a highly conductive network to promote electron transport. Moreover, the presence of oxygen vacancies, evidenced by both EPR and XPS analyses, is believed to enhance electronic conductivity and lower the energy barrier for Na^+^ diffusion, thereby facilitating faster charge transport kinetics. Similar synergistic effects involving nanoconfinement, conductive carbon frameworks, and vacancy engineering have been demonstrated in previous studies [46,47].

The above results confirm that the ov-SnO_2_/rGO@N-CNFs composite exhibits reduced charge-transfer resistance and superior sodium ion transport capability. These electrochemical advantages originate from the rationally engineered multi-level architecture of the composite. Specifically, the GO-induced redox synthesis enables the formation of ultra-small SnO_2_ dots (~2.3 nm) with abundant oxygen vacancies, which provide numerous active sites and lower diffusion barriers for Na^+^ insertion/extraction. The rGO sheets offer a conductive framework to enhance electron transport, while the N-CNFs matrix suppresses SnO_2_ agglomeration and mitigates volume expansion through mechanical confinement. This synergistic design not only stabilizes the electrode structure during long-term cycling but also facilitates rapid charge transfer and ion diffusion. Consequently, the unique structural features of ov-SnO_2_/rGO@N-CNFs lead to significantly improved sodium storage performance, including substantial capacity retention, superior high-rate performance, and exceptional cycling durability.

## 3. Experimental

### 3.1. Preparation of Ov-SnO_2_/rGO@N-CNFs

Polyacrylonitrile (PAN) was sourced from Guangzhou Quanjin Trade in Guangzhou, China, and N, N-dimethylformamide (DMF, 99.5 wt%) was obtained from Aladdin Chemical Co. Ltd in Shanghai, China. Pristine SnO_2_ was obtained from Aladdin Chemical Co. Ltd in China. SnCl_2_·2H_2_O was bought from Sinopharm Chemical Reagent Co., Ltd in Shanghai, China. Graphene oxide was prepared in the laboratory. All reagents were of analytical grade and were used without further purification.

GO was synthesized from natural graphite powder following a previously reported method [21]. Typically, GO and SnCl_2_·2H_2_O (1:14, mass ratio) were dissolved in 10 mL of DMF and stirred at 40 °C for 6 h to obtain SnO_2_/rGO-DMF. Then, 1.2 g of PAN was added and stirred for another 6 h to achieve a uniform precursor solution. The solution was electrospun using a 20 G needle (1.1 mm inner diameter) at 16 kV, with a flow rate of 0.08 mL min^−1^ and a 12 cm distance from the rotating collector (300 rpm), under 25 °C and 40% humidity, forming an as-spun film (ov-SnO_2_/rGO/PAN). The film was pre-oxidized at 220 °C for 1 h (2 °C min^−1^) and then carbonized at 600 °C for 2 h (1 °C min^−1^) under nitrogen to obtain ov-SnO_2_/rGO@N-CNFs. The pre-oxidation temperature of 220 °C was chosen based on established protocols for stabilizing PAN-based nanofibers, which undergo cyclization and oxidation reactions necessary for maintaining their fibrous morphology during carbonization. Carbonization at 600 °C was chosen as it allows the formation of a flexible, self-supporting carbon nanofiber membrane. This temperature provides sufficient structural integrity and continuity for the membrane to maintain freestanding form, while preserving nitrogen content and preventing excessive SnO_2_ crystallization or reduction. However, graphitization at this temperature may restrict the intrinsic conductivity; increasing the carbonization temperature can enhance graphitization and thereby improve electrical conductivity. Nonetheless, such higher temperatures often compromise the mechanical flexibility of the resulting carbon nanofibers, rendering them brittle and susceptible to fragmentation. In contrast, carbonization at 600 °C effectively preserves the structural flexibility of the self-supporting membrane, which is essential for practical device integration and long-term cycling stability. For comparison, Sn@N-CNFs were prepared following an identical procedure without the addition of GO. To investigate the influence of the solvent on SnO_2_ dispersion, SnO_2_/rGO-H_2_O was also prepared by replacing DMF with water. SnO_2_/rGO-DMF denotes the intermediate product after the redox reaction of GO and SnCl_2_·2H_2_O in DMF, which was collected before electrospinning and carbonization. To evaluate the protective role of the N-doped carbon nanofibers, SnO_2_/rGO-DMF was annealed at 600 °C for 2 h under nitrogen (2 °C min^−1^). The carbonized counterpart is labeled as Carbonized-SnO_2_/rGO-DMF.

### 3.2. Material Characterization

The phase composition of the samples was examined via X-ray diffraction (X’Pert PRO MPD, PANalytical, Malvern, UK) with Cu Kα radiation (λ = 0.15418 nm), scanning a 2θ angle from 5° to 75°. Morphological and microstructural characteristics were investigated using scanning electron microscopy (Hitachi S-4800, Tokyo, Japan) and transmission electron microscopy (JEOL-2100F, JEOL Ltd., Tokyo, Japan, 200 kV). Elemental distribution was analyzed through energy-dispersive X-ray spectroscopy (EDX) on the same TEM instrument. Chemical states were determined by X-ray photoelectron spectroscopy (Escalab 250Xi, Thermo Fisher Scientific, Shanghai, China) with an Mg Kα (1486.6 eV) excitation source. Electron paramagnetic resonance (Bruker A300, Bruker, Beijing, China) spectra were recorded at room temperature (298 K) with a 100 kHz modulation frequency. Additionally, elemental composition was quantified by inductively coupled plasma-atomic emission spectroscopy (ICP-AES, 730ES, Agilent Technologies, Qingdao, China).

### 3.3. Electrochemical Measurements

CR2032 coin cells were assembled in an argon-filled glove box (H_2_O and O_2_ levels < 0.1 ppm) at ambient temperature to assess the electrochemical behavior. The ov-SnO_2_/rGO@N-CNFs composite served as a freestanding anode for SIBs, eliminating the need for additional binders, conductive additives, or metal current collectors. Circular electrodes (12 mm diameter) were prepared from the flexible membranes for use as working electrodes, paired with sodium metal counter electrodes. The active material (ov-SnO_2_/rGO@N-CNFs) exhibited a mass loading of approximately 1.1 mg·cm^−2^. The electrochemical cells employed Celgard 2300 separators, with an electrolyte formulation of 1 M NaClO_4_ in a binary solvent system comprising ethylene carbonate and propylene carbonate (EC:PC = 2:1 by volume).

The electrochemical performance was evaluated through galvanostatic cycling tests performed on a LAND CT2001A test system, with an operational potential range of 0.01–3.00 V relative to Na^+^/Na. Additionally, impedance characteristics were analyzed via electrochemical impedance spectroscopy (EIS) using an Ametek PARSTAT4000 (Beijing, China) instrument, scanning frequencies from 10^6^ to 10^−1^ Hz.

## 4. Conclusions

In conclusion, this work demonstrates a novel anode design strategy that synergistically combines GO-induced oxygen-vacancy engineering with N-doped carbon nanofiber confinement to address the critical challenges of SnO_2_-based materials for sodium-ion batteries. The synergistic integration of oxygen-deficient SnO_2_ dots, conductive rGO network, and protective N-CNFs establishes a hierarchical architecture to achieve additional sodium intercalation sites, enhanced Na^+^/electron transport, and buffered SnO_2_ aggregation. This distinctive hierarchical architecture enables outstanding performance, delivering both high reversible capacity (351 mAh^−1^ at 0.1 A g^−1^) and exceptional long-term cycling stability (177 mAh g^−1^ after 8000 cycles at 5 A g^−1^). Our findings not only present a high-performance SnO_2_-based anode material but also establish a generalizable design principle combining defect engineering with structural optimization, opening new possibilities for developing advanced electrode materials in next-generation energy storage systems.

## Data Availability

The original contributions presented in this study are included in the article and Supplementary Materials; further inquiries can be directed to the corresponding author.

## Supplementary Materials

The following supporting information can be downloaded at: https://www.mdpi.com/article/10.3390/molecules30153203/s1, Figure S1. XRD patterns of pristine SnO_2_, SnO_2_/rGO-H_2_O, and SnO_2_/rGO-DMF. Figure S2. SEM image of ov-SnO_2_/rGO@N-CNFs. Figure S3. XRD patterns of Sn@N-CNFs, ov-SnO_2_/rGO@N-CNFs, SnO_2_/rGO-DMF, and carbonized-SnO_2_/rGO-DMF. Figure S4. TEM images of (a) ov-SnO_2_/rGO@N-CNFs and (b) Sn@N-CNFs after 2000 cycles at 1 A g^−1^. Figure S5. Cycling performance of rGO@N-CNFs at 0.1 A g^−1^. Figure S6. Real parts of the impedance (Z’) versus the reciprocal square root of angular frequency (ω) in the low frequency region of ov-SnO_2_/rGO@N-CNFs after 400, 800, and 1000 cycles. Table S1. ICP-AES testing results of ov-SnO_2_/rGO@N-CNFs. Table S2. Comparison of the theoretical and testing capacities of ov-SnO_2_/rGO@N-CNFs at the current density of 0.1 A g^−1^. Table S3. Electrochemical impedance parameters of the ov-SnO_2_/rGO@N-CNFs electrode after 0, 200, 400, 600, 800, and 1000 cycles.
