# Supplementary material for: Oxygen-Vacancy Engineered SnO2 Dots on rGO with N-Doped Carbon Nanofibers Encapsulation for High-Performance Sodium-Ion Batteries
Source: Molecules. 2025 Jul 30;30(15):3203. doi: 10.3390/molecules30153203 (PMC12348730; doi:10.3390/molecules30153203)
Supplement: Supplementary file 1 [file molecules-30-03203-s001.zip › molecules-3754239-supplementary.pdf]

Supplementary Information

Oxygen-Vacancy Engineered SnO_2 Dots on rGO with N-Doped Carbon Nanofibers Encapsulation for High-Performance Sodium-Ion Batteries

Yue Yan [1], Bingxian Zhu [1], Zhengzheng Xia [1], Hui Wang [1], Weijuan Xu [1], Ying Xin [1], Qingshan Zhao [1,*] and Mingbo Wu [1,2]

[1] State Key Laboratory of Heavy Oil Processing, Shandong Key Laboratory of Advanced Electrochemical Energy Storage Technologies, College of Chemistry and Chemical Engineering, China University of Petroleum (East China), Qingdao 266580, China; 15940535308@163.com (Y.Y.); zhubingxian0909@163.com (B.Z.); xiazz2021@163.com (Z.X.); 17613877583@163.com (H.W.); 19861453181@163.com (W.X.); 18364538700@163.com (Y.X.); wumb@upc.edu.cn (M.W.)

[2] College of Chemical Engineering, Qingdao University of Science & Technology, Qingdao 266100, China

* Correspondence: qszhao@upc.edu.cn

Calculation of the D_{Na+}

The D_{Na+} of ov-SnO_2/rGO@N-CNFs according to the EIS results based on the following equations:

$$D_{Na+}=0.5 \times (nF)^{-4}C^{-2}\sigma^{-2}R^2T^2A^2 \quad (S1)$$

where R is the gas constant, T is the absolute temperature, A is the electrochemical reaction area, C is the concentration of Na^+ in the electrolyte (~1.0×10^{-3} mol cm^{-3}), and σ is the Warburg factor which has relationship with Z′:

$$Z'=R_D + R_C + \sigma\omega^{-1/2} \quad (S2)$$

Figure S6 shows the relationship between Z' and square root of frequency ($\omega^{-1/2}$) in the low-frequency region. The diffusion coefficient of sodium ion is calculated based on equation (S1) and equation (S2). The sodium ion diffusion coefficients of ov-SnO_2/rGO@N-CNFs after 400 C, 800 C, 1000 C are calculated to be 1.32×10^{-13} cm^2 s^{-1}, 1.55×10^{-13} cm^2 s^{-1} and 3.02×10^{-13} cm^2 s^{-1}, respectively.

The theoretical and testing capacities of *ov*-SnO_2/rGO@N-CNFs

Since the theoretical capacities of SnO_2 are 667 mAh g^{-1}, and rGO@N-CNFs provides a capacity of 214 mAh g^{-1} at 0.1A g^{-1} according to the testing result in Fig. S5. The SnO_2 loadings of ov-SnO_2/rGO@N-CNFs were tested by inductively coupled plasma-atomic emission spectrometry (ICP-AES), as listed in Table S1. The mass ratios of Sn are determined to be 21.68 wt%. Accordingly, the mass percentage of SnO_2 in the ov-SnO_2/rGO@N-CNFs composites are calculated to be 27.52 wt%. Thus, the theoretical capacities of ov-SnO_2/rGO@N-CNFs are calculated by the equation of $C_{ov\text{-}SnO_2/rGO@N\text{-}CNFs} = C_{SnO_2} \times \%_{mass\ of\ SnO_2} + C_{rGO@N\text{-}CNFs} \times \%_{mass\ of\ rGO@N\text{-}CNFs}$, which are determined to be 340.0 mAh g^{-1} (details listed in Table S2).

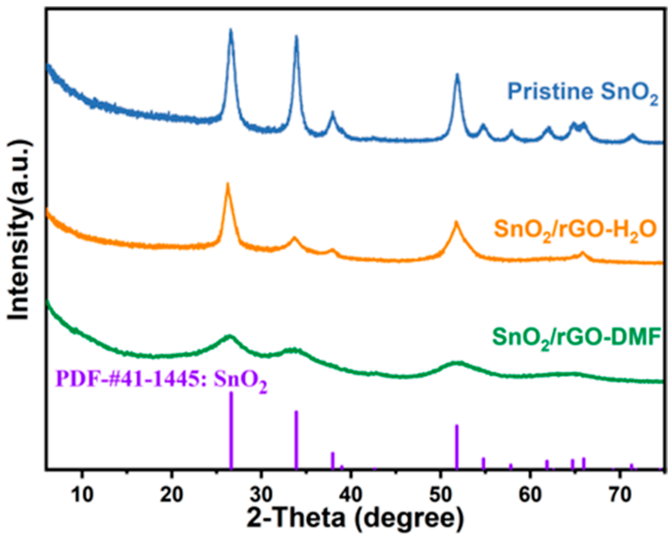

Figure S1. XRD patterns of pristine SnO_2, SnO_2/rGO-H_2O, and SnO_2/rGO-DMF.

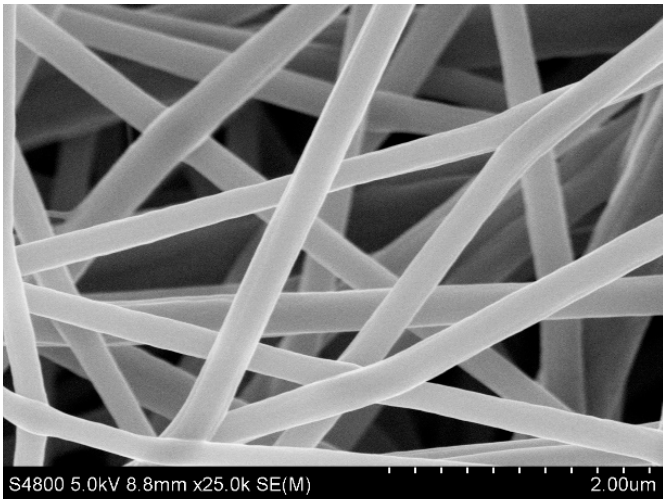

Figure S2. SEM image of *ov*-SnO_2/rGO@N-CNFs.

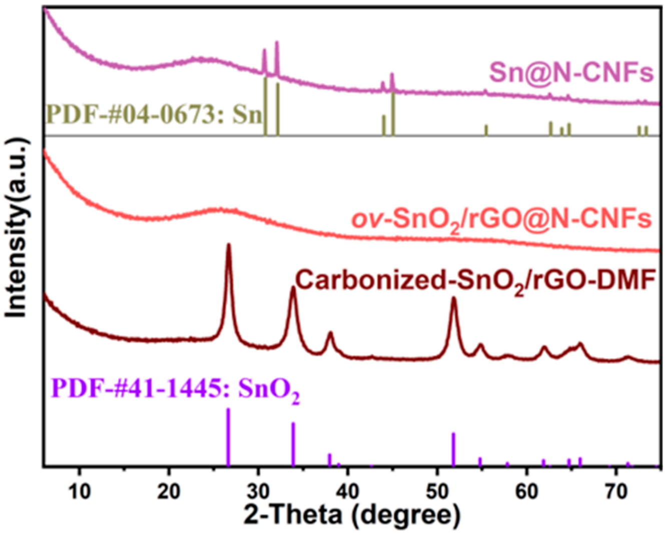

Figure S3. XRD patterns of Sn@N-CNFs, *ov*-SnO_2/rGO@N-CNFs, SnO_2/rGO-DMF, and carbonized-SnO_2/rGO-DMF .

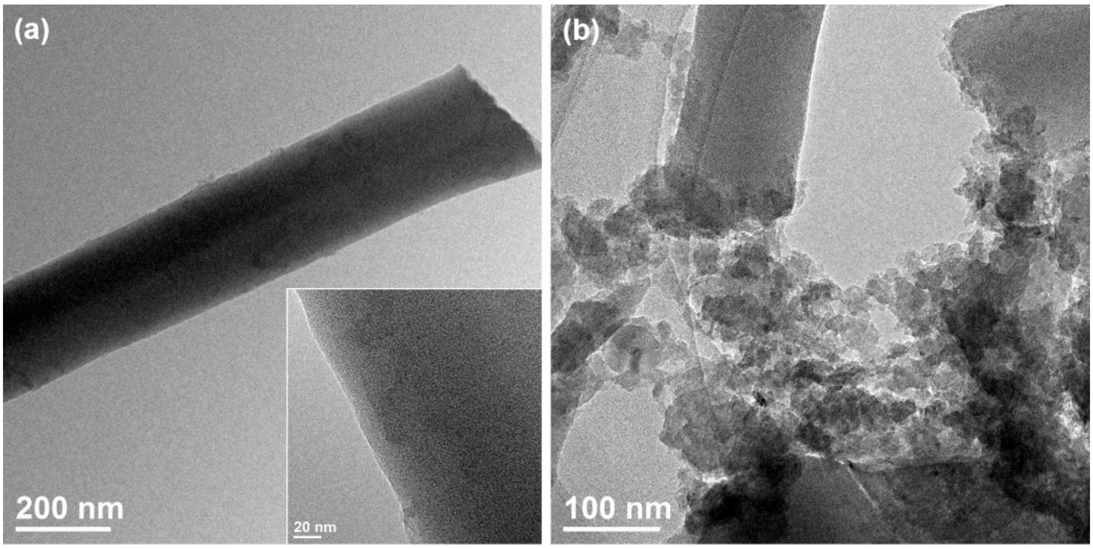

Figure S4. TEM images of (a) ov-SnO_2/rGO@N-CNFs and (b) Sn@N-CNFs after 2000 cycles at 1A g^{-1}.

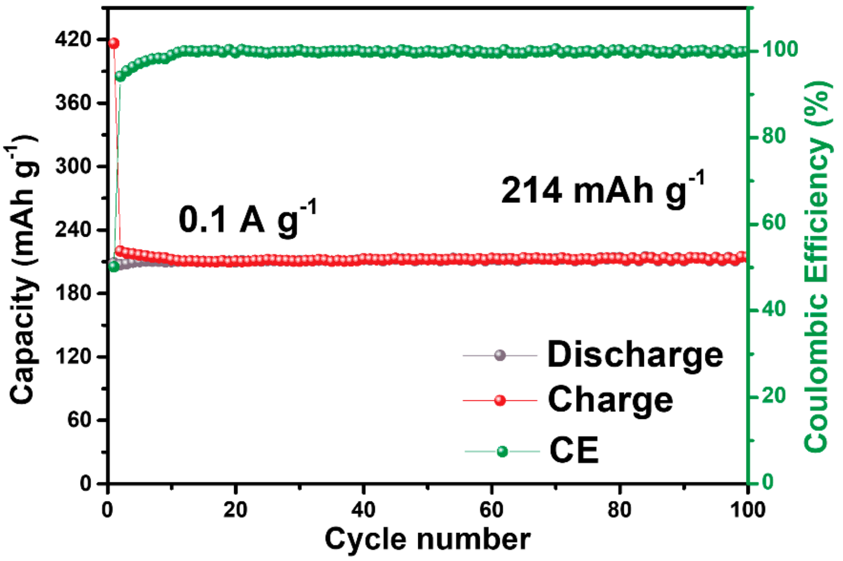

Figure S5. Cycling performance of rGO@N-CNFs at 0.1 A g^{-1}.

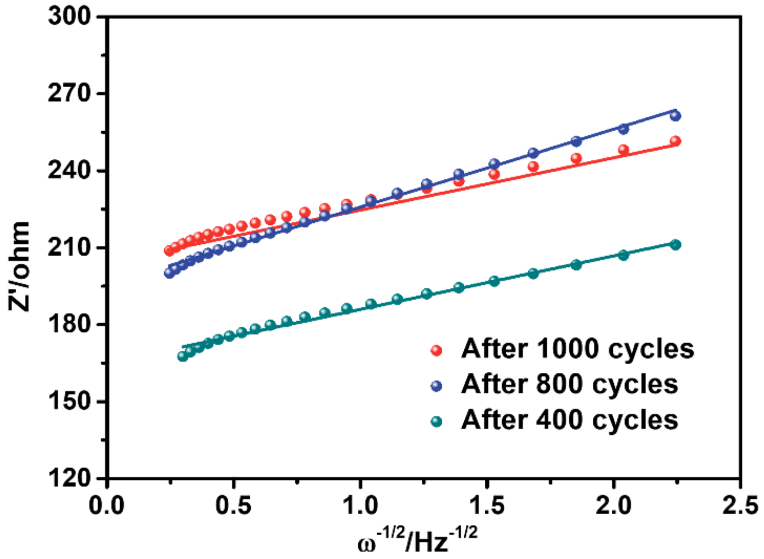

Figure S6. Real parts of the impedance (Z′) versus the reciprocal square root of angular frequency (ω) in the low frequency region of ov-SnO_2/rGO@N-CNFs after 400, 800 and 1000 cycles.

Table S1. ICP-AES testing results of *ov*-SnO_2/rGO@N-CNFs.

Sample	Element	Mass ratios
ov-SnO_2/rGO@N-CNFs	Sn	21.68 wt%

Table S2. Comparison of the theoretical and testing capacities of ov-SnO_2/rGO@N-CNFs at the current density of 0.1 A g^{-1}.

Metal oxide	SnO_2
Theoretical capacity (mAh g^{-1})	667
Mass percentage of SnO_2 (%)	27.52
Capacity of rGO@N-CNFs (mAh g^{-1})	214.0
Mass percentage of rGO@N-CNFs (%)	72.48
Theoretical capacity of ov-SnO_2/rGO@N-CNFs (mAh g^{-1})	340.7
Testing capacity of ov-SnO_2/rGO@N-CNFs (mAh g^{-1})	351

Table S3. Electrochemical impedance parameters of the ov-SnO_2/rGO@N-CNFs electrode after 0, 200, 400, 600, 800 and 1000 cycles.

Cycle number	R_{ct} (Ω)	R_s (Ω)
0	912.4	22.4
200	237.6	16.3
400	136.3	4.6
600	148.2	2.3
800	196.3	3.1
1000	211.4	3.7
